# Factors Associated with Vitamin D Deficiency and Inadequacy among Women of Childbearing Age in the United States

**DOI:** 10.5402/2012/691486

**Published:** 2012-03-04

**Authors:** Guixiang Zhao, Earl S. Ford, James Tsai, Chaoyang Li, Janet B. Croft

**Affiliations:** ^1^Division of Adult and Community Health, National Center for Chronic Disease Prevention and Health Promotion, Centers for Disease Control and Prevention, Atlanta, GA 30341, USA; ^2^Division of Behavioral Surveillance, Public Health Surveillance Program Office, Office of Surveillance, Epidemiology and Laboratory Services and Centers for Disease Control and Prevention, Atlanta, GA 30341, USA

## Abstract

*Objective*. To examine the prevalence and correlates of vitamin D deficiency and inadequacy among US women of childbearing age. *Methods*. Data from 1,814 female participants (20–44 y) in the 2003–2006 NHANES were analyzed to estimate the age-adjusted prevalence and prevalence ratios with 95% confidence intervals (CIs) for vitamin D deficiency (defined as serum 25-hydroxyvitamin D [25(OH)D] <12.0 ng/mL) and inadequacy (defined as 25(OH)D: 12.0–<20.0 ng/mL). *Results*. The age-adjusted prevalence was 11.1% (95% CI: 8.8–14.0%) for vitamin D deficiency and 25.7% (95% CI: 22.3–29.5%) for vitamin D inadequacy. Race/ethnicity other than non-Hispanic white and obesity were associated with increased risks, whereas dietary supplement use, milk consumption of ≥1 time/day, and potential sunlight exposure during May-October were associated with decreased risks for both vitamin D deficiency and inadequacy (*P* < 0.05). Current smoking and having histories of diabetes and cardiovascular disease were also associated with an increased risk for vitamin D deficiency (*P* < 0.05). *Conclusions*. Among women of childbearing age, periconceptional intervention programs may focus on multiple risk factors for vitamin D deficiency and inadequacy to ultimately improve their vitamin D nutrition.

## 1. Introduction

Vitamin D, a lipid-soluble vitamin or steroid prehormone, is known to play an important role in bone metabolism through regulation of calcium and phosphate homeostasis. During the past few years, the influence of vitamin D on nonskeletal biologic processes has been expanded to a wide array of health outcomes including cardiovascular disease, insulin resistance and diabetes, cancer, respiratory infection, innate and adaptive immunity, and both cardiovascular and all-cause mortality [1–9].

For women of childbearing age, maternal vitamin D malnutrition imposes multiple health impacts on both women and their offspring [10–12]. Adequate vitamin D concentrations during pregnancy help ensure appropriate maternal and fetal calcium homeostasis and bone metabolism [11, 12]. Consequently, vitamin D deficiency during pregnancy has been associated with impaired fetal skeletal formation causing infant rickets and reduced bone mass [13–15]. In addition, low maternal vitamin D concentrations have been associated with spontaneous preterm birth and impaired embryonic and fetal development (including skeletal, brain, and immunological function development) resulting in small-for-gestational-age infants [16–18]. Moreover, childhood vitamin D deficiency, which is strongly associated with maternal vitamin D deficiency [19, 20], has been linked to increased risk for multiple conditions including respiratory infection [21], asthma [22, 23], type 1 diabetes [24], and schizophrenia [25]. For maternal health, a low vitamin D level impairs maternal skeletal preservation and is associated with increased risks for osteomalacia, osteoporosis, miscarriage, preeclampsia, gestational diabetes, and bacterial vaginosis [10, 26–28].

Limited evidence has shown that pregnant women and neonates residing in the northern USA are at high risk for vitamin D insufficiency regardless the use of vitamin supplementation [10, 29]. A recent study reported that about 69% of pregnant women and 78% of nonpregnant women in the USA had serum 25-hydroxyvitamin D [25(OH)D] concentrations below 30.0 ng/mL [30]. In those studies, however, the 25(OH)D cut-point values defined as indicative of deficiency or insufficiency varied significantly and was lack of consensus. The Institute of Medicine (IOM) recently released new definitions for vitamin D deficiency (i.e., 25(OH)D <12 ng/mL (30 nmol/L)) and inadequacy (i.e., 25(OH)D 12–<20 ng/mL (30–<50 nmol/L))[31]. Thus, by using data from a large, nationally representative sample of US women, the present study aimed to (1) examine the prevalence of vitamin D deficiency and inadequacy among women of childbearing age based on the new IOM definitions and (2) identify potential factors including sociodemographics, dietary and lifestyle-related behaviors, health conditions, and healthcare utilization that may have predisposed women to increased risk for vitamin D deficiency or inadequacy. We believe this information is valuable for women's health care providers including obstetricians and gynecologists to provide better counseling for women of childbearing age concerning their need for vitamin D.

## 2. Methods

### 2.1. Study Design

Data for this analysis came from the 2003–2006 National Health and Nutrition Examination Survey (NHANES), which uses a multistage stratified sampling design to collect data from the noninstitutionalized civilian US population on a 2-year cycle basis. Survey participants were initially interviewed at home and then invited to a mobile examination center (MEC), where they received various examinations and provided blood samples for laboratory tests. In the NHANES 2003–2006, all procedures involving human subjects were approved by the Research Ethics Review Board of the National Center for Health Statistics and written informed consent was obtained from all participants. Details about the NHANES survey design and methods are reported elsewhere [32].

### 2.2. Participants and Measurements

We examined interview and laboratory data from female participants aged 20–44 years. The pregnancy status of a woman was determined as (1) current pregnancy which was confirmed either by a positive laboratory pregnancy test or by a self-reported pregnancy at the time of MEC exam, (2) nonpregnant with a history of pregnancy, and (3) never pregnant. Participants whose pregnancy status could not be ascertained were excluded from our analyses.

Participants' serum specimens were frozen and stored at <–70°C until analysis. Serum concentrations of 25(OH)D were measured using the DiaSorin radioimmunoassay procedure (DiaSorin Corporation 25(OH)D ^125^I RIA kit, DiaSorin Corporation, Stillwater, MN), which has been described in detail in the laboratory procedure manuals of the NHANES website [32]. The 25(OH)D assay has a sensitivity of 1.5 ng/mL; the coefficient of variation ranged from 8.9% for NHANES 2003-2004 participants to 9.9% for NHANES 2005-2006 participants.

The demographic covariates in our analyses were participants' age (20–27 years, 28–34 years, and 35–44 years), race/ethnicity (non-Hispanic white, non-Hispanic black, Mexican American, and other), and education level (< high school diploma, high school graduate or equivalent, and > high school diploma). Major food sources of vitamin D intake were assessed by asking participants how often in the previous 30 days they had consumed milk (never, >0 to <1 time/day, and ≥1 time/day), or they had eaten fish (never, >0 to <2 times/week, and ≥2 times/week). Potential sunlight exposure was assessed using the 6-month time period during which they were examined (categorized as November 1 through April 30 and May 1 through October 31). Dietary supplement use was assessed by asking participants whether they had taken any dietary supplements during the previous month (yes, no). Body mass index (BMI) for currently pregnant women was calculated from self-reported, prepregnancy weight and height, and the BMI for nonpregnant women was calculated from measured weight and height following standardized protocol and instruments (<25.0, 25.0–29.9, and ≥30.0 kg/m^2^). Smoking status was categorized as current smokers (participants who had smoked at least 100 cigarettes during their lifetime and were still smoking) and nonsmokers (participants who had smoked less than 100 cigarettes during their lifetime or those who had smoked at least 100 cigarettes during their lifetime but had stopped). Physical activity was calculated as an average daily metabolic equivalent task (MET)-hour index that summed transportation, household, and leisure-time physical activity, and participants were dichotomized as having physical activity (MET-hour index >0) and physically inactive (MET-hour index = 0). Alcohol consumption was categorized as any alcohol users (participants who had an average daily drinks of >0 during the past 12 months) and abstainers (for whom the average daily drink was equal to 0 in the past 12 months). Healthcare access was assessed by asking participants: (1) whether they were covered by health insurance or some other kind of health care plan including those obtained through employment or purchased directly as well as government programs like Medicare and Medicaid that provide medical care or help pay medical bills (yes, no), and (2) whether, during the past 12 months, they had seen a doctor or other health care professional about their health at a doctor's office, a clinic, hospital emergency room, at home or some other place (yes, no).

The medical conditions assessed in the study were hypertension, physician diagnosed diabetes and cardiovascular disease (including coronary heart disease, angina pectoris, myocardial infarction, and stroke), and current asthma. The mean systolic and diastolic blood pressure (SBP and DBP) were established by using the average of the last two readings of SBP or DBP for participants whose blood pressure was measured three or four times, or using the last reading for participants whose blood pressure was measured twice, or using the only reading for participants whose blood pressure was measured only once. Participants who reported taking antihypertension medications or who had SBP ≥140 mmHg or DBP ≥90 mmHg were defined as having hypertension [33]. Physician-diagnosed diabetes or cardiovascular disease were assessed by asking participants whether they had ever been told by a healthcare professional that they had these conditions. Current asthma was assessed by asking participants whether they had ever been told that they had asthma and still had it during the survey.

### 2.3. Statistical Analysis

Of 2,443 female NHANES participants aged 20–44 years, we excluded 349 women who responded “do not know/not sure,” refused to answer, or had missing responses to the question regarding their pregnancy status. After further excluding those who had missing values for their 25(OH)D concentrations or other study covariates, 1,814 women remained in our analyses. The estimates for the mean concentration of serum 25(OH)D and the prevalence of vitamin D deficiency and inadequacy were weighted and age-adjusted to the 2000 US female population aged 20–44 years. We used log-linear regression models with robust variance estimator to estimate unadjusted and multivariate-adjusted prevalence ratios with 95% confidence intervals (CIs). We used a Bonferroni corrected *P* value for multiple comparisons and used SUDAAN (Software for the Statistical Analysis of Correlated Data, Release 9.0, Research Triangle Institute, Research Triangle Park, NC) to account for the complex sampling design.

## 3. Results

Of all study participants, 435 were currently pregnant and 1,034 reported having a history of pregnancy. Their mean age was 32.6 years (95% CI: 32.2–33.1). Approximately 66.8% of women were non-Hispanic white, 12.1% non-Hispanic black, 10.0% Mexican American, and 11.0% in other racial/ethnic group. About 14.6% of women had less than a high school education, 21.2% were high school graduates or equivalents, and 64.2% attained an educational level of greater than a high school diploma.

**Figure 1:**
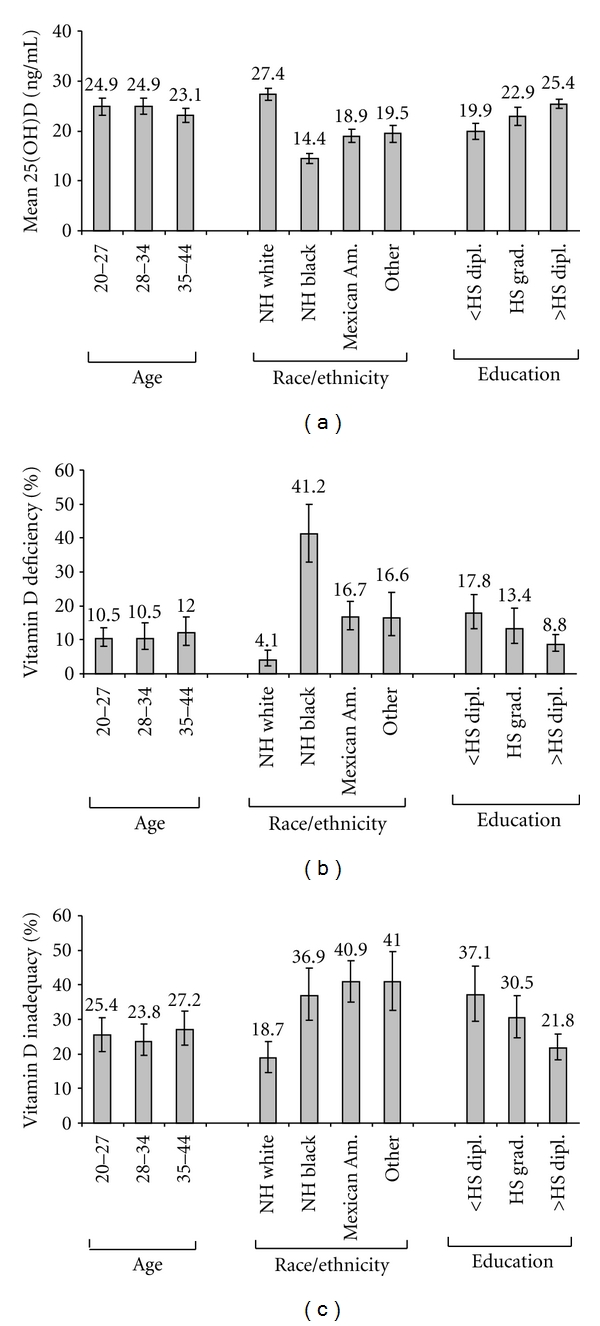
Age-adjusted mean concentrations (with 95% CIs) of serum 25(OH)D (a) and the age-adjusted prevalence (with 95% CIs) of vitamin D deficiency (b) and inadequacy (c) by demographic characteristics among women of childbearing age (20–44 years, *N* = 1,814) in the United States, NHANES 2003–2006.

**Figure 2:**
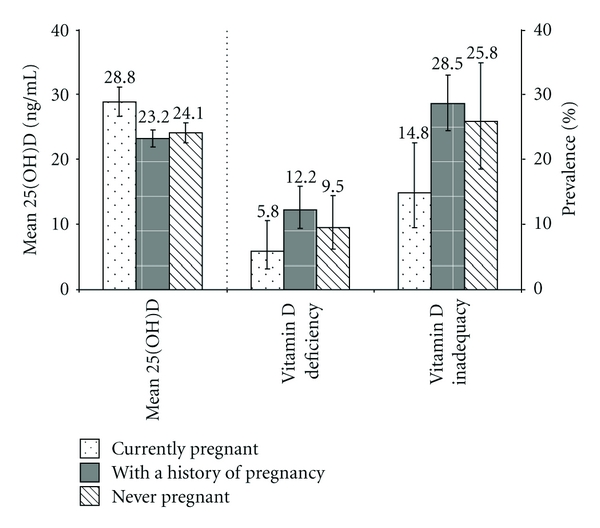
Age-adjusted mean concentrations (with 95% CIs) of serum 25(OH)D and the age-adjusted prevalence (with 95% CIs) of vitamin D deficiency and inadequacy by pregnancy status among women of childbearing age (20–44 years, *N* = 1,814) in the United States, NHANES 2003–2006.

The mean 25(OH)D concentration was 24.1 ng/mL (95% CI: 23.0–25.1 ng/mL), the prevalence of vitamin D deficiency was 11.1% (95% CI: 8.8–14.0%), and the prevalence of vitamin D inadequacy was 25.7% (95% CI: 22.3–29.5%). These measures did not differ significantly by age (Figure 1). By race/ethnicity, the mean 25(OH)D concentration was the highest among non-Hispanic white women and the lowest among non-Hispanic black women (Bonferroni corrected  *P* < 0.008, Figure 1(a)). Conversely, the prevalence of vitamin D deficiency and inadequacy was lowest among non-Hispanic white women (Figures 1(b) and 1(c)) and the prevalence of vitamin D deficiency was the highest among non-Hispanic black women (Figure 1(b)). By educational level, the mean 25(OH)D concentration increased linearly (Figure 1(a)), whereas the prevalence of vitamin D deficiency and inadequacy decreased linearly (*P* < 0.001 for linear trends, Figures 1(b) and 1(c)). By pregnancy status, the mean 25(OH)D concentration was higher among pregnant women than among those in the other two groups (Bonferroni corrected *P* < 0.017, Figure 2); the prevalence of vitamin D deficiency and inadequacy was lower among pregnant women than among women with a history of pregnancy (Bonferroni corrected *P* < 0.017).

By race/ethnicity, the multivariate-adjusted prevalence ratio for vitamin D deficiency was 8.3 times as high among non-Hispanic black women, 4.1 times as high among Mexican American women, and 5.4 times as high among women in other racial/ethnic groups as among non-Hispanic white women (*P* < 0.001 for all, Table 1); the adjusted prevalence ratio for vitamin D inadequacy was also significantly higher in these groups (1.8–2.8 times as high as in non-Hispanic white women, *P* < 0.001 for all, Table 1). By BMI status, the adjusted prevalence ratios for vitamin D deficiency and inadequacy were 1.7 times and 1.9 times as high, respectively, among women who were obese (BMI ≥30.0 kg/m^2^) as among women with a normal BMI (*P* < 0.001 for both). Additionally, the adjusted prevalence ratio for vitamin D deficiency was 1.5 times as high among women with diabetes, 2.3 times as high among women with cardiovascular disease, and 1.4 times as high among women who were current smokers as among their respective counterparts.

In contrast, milk consumption ≥1 time/day, dietary supplement use, and potential sunlight exposure during May-October were significantly and independently associated with lower prevalence ratios of vitamin D deficiency and inadequacy (*P* < 0.05 for all, Table 1). Current pregnancy was marginally associated with a lower prevalence ratio of vitamin D inadequacy (*P* = 0.051). Although education of > high school diploma, physical activity, any alcohol use, insurance coverage, and having had a health care visit were all associated with decreased likelihoods of vitamin D deficiency and inadequacy in unadjusted models, they were not significant contributors to the full models when controlling for all study covariates. 

## 4. Discussion 

Our study on a large, nationally representative sample demonstrated that applying the new IOM definitions predicted a lower prevalence of vitamin D deficiency and inadequacy than that was reported previously [29, 30]. Our results showed that more than one-third of women of childbearing age had their vitamin D status below an optimal level (i.e., ≥20.0 ng/mL [31]) in the United States. More importantly, we found that multiple factors were independently associated with suboptimal levels of vitamin D among these women. 

Significant changes in maternal vitamin D and calcium metabolism occur during pregnancy. For example, a study has reported that serum concentrations of 1,25-dihydroxyvitamin D [1,25(OH)_2_D], the active form of vitamin D, increased by 50–100% from the nonpregnant state to the second to third trimesters of the pregnancy [34]. A longitudinal study conducted in Caucasian women of the United Kingdom showed that plasma 25(OH)D concentrations were lower among pregnant women than among nonpregnant controls [35], a finding that may be largely explained by increased fetal demand for this essential nutrient which is almost entirely dependent on vitamin D from the mother [18, 19]. However, results from other cross-sectional studies have shown, similar to our findings, that pregnant women have a higher mean 25(OH)D concentration than nonpregnant women [29, 30, 36]. The higher 25(OH)D concentrations among pregnant women are likely due to higher rates of dietary supplement use (i.e., 83.4% versus 47.3%) and milk consumption of at least 1 time/day (i.e., 63.8% versus 41.4%) among pregnant women than among nonpregnant women. 

Our study showed that multiple factors were associated with suboptimal vitamin D status in women of childbearing age. The significant racial/ethnic disparities that we found in the prevalence of vitamin D deficiency/inadequacy were in agreement with those reported previously both in the general population as well as in pregnant women, showing non-Hispanic whites had the highest adjusted mean 25(OH)D concentration, followed by Mexican Americans, and non-Hispanic blacks had the lowest [30, 36–38]. Our results further demonstrated that, among US women of reproductive age, those who were non-Hispanic black, Mexican American, or of other racial/ethnic groups were all at a higher risk, to the similar magnitude, for vitamin D deficiency and inadequacy than non-Hispanic white women. 

In the human body, about 50–90% of vitamin D comes from the biosynthesis of vitamin D3 (cholecalciferol) in the skin from 7-dehydrocholesterol that requires sunlight (ultraviolet radiation), and the remainder comes from a limited number of foods (mainly fatty fish, eggs, and liver as well as foods fortified with vitamin D such as margarine, cereals, and milk products) and from dietary supplements. A previous study based on data from the third NHANES (1988–1994) reported that seasonality, milk consumption, and dietary supplement use were significant and independent determinants of hypovitaminosis D in women of childbearing age [39], which is consistent with the findings of the present study. Fish consumption can be a major source of vitamin D intake. Lym and Joh recently reported that frequent fish consumption was associated with a higher vitamin D level among Korean men [40]. Brock et al. also reported that a high fish intake was associated with 25(OH)D ≥25 nmol/L (10 ng/mL) in Finland male smokers exposed to negligible solar UV light in winter [41]. In the present study, however, this relationship was not significant in women of childbearing age. Thus, future research on the role of fish consumption in improving vitamin D nutrition is warranted. 

Low vitamin D levels are linked to increased risk for high blood pressure, insulin resistance and diabetes, and cardiovascular disease in the general population [2, 6–9]. Similarly, among women of childbearing age, we found that having a history of diabetes or cardiovascular disease was significantly associated with vitamin D deficiency (but not vitamin D inadequacy) independent of other factors. Thus, women with these conditions should be assessed and counseled for their vitamin D status during their routine health care visits. 

Multiple health-related behavioral factors have been shown to affect pregnancy outcomes. For example, physical inactivity in mothers is associated with increased risks for obesity and obesity-related chronic conditions such as diabetes mellitus and hypertension, and pregnant women who are obese are at increased risk for miscarriage, pregnancy-induced hypertension and preeclampsia, gestational diabetes, and thromboembolism, and at an increased risk of having children with macrosomia, spontaneous intrauterine demises, or delivered by Cesarean section which carries an increased risk for wound infection [42–44]. Maternal alcohol use and cigarette smoking have been associated with a higher rate of infertility, spontaneous abortion or preterm birth, fetal alcohol syndrome (characterized by growth deficiencies, central nervous system impairment, behavioral disorders, and impaired intellectual devolvement with lifelong implication), and facial dysmorphia [45–48]. Thus, maintaining healthy lifestyles is especially important for women of childbearing age. Previous studies have consistently reported low serum vitamin D concentrations in people with a high BMI [38, 49, 50], results consistent with our finding that BMI of ≥ 30.0 kg/m^2^ was an independent predictor of vitamin D deficiency and inadequacy among women of childbearing age. Smoking has been associated with a low bone mass and an increased risk for osteoporotic fracture [51]. Brot et al. further reported that middle-aged women who were current smokers had significantly reduced levels of 25(OH)D, 1,25(OH)_2_D and parathyroid hormone [52]. Our results also showed that smoking was independently associated with vitamin D deficiency in women of childbearing age. However, we found that other behavioral factors such as physical activity and alcohol drinking were not significant determinants of vitamin D status. 

Nutritional assessment, education, and counseling are important components of periconception care. In the present study, although the prevalence of vitamin D deficiency and inadequacy decreased linearly across educational levels, education was not a significant contributor to the models after controlling for potential confounders. Also, we found that increasing use of health care services was not a significant determinant of vitamin D status either. However, these findings should be viewed with caution because the effectiveness of nutritional education and counseling was not assessed in this study. 

This study has several limitations. First, the causal relationships between factors and vitamin D deficiency/inadequacy cannot be established from our cross-sectional study design. Second, the drift in serum 25(OH)D measurements over the time has been reported. However, we did not find significant differences in the prevalence of vitamin D deficiency and inadequacy between the NHANES 2003-2004 and 2005-2006 survey periods, suggesting that our results may not have been affected in a meaningful way by the variability of serum 25(OH)D assay [53]. 

In conclusion, our results demonstrate that achieving an optimal vitamin D level remains a distant target for women of childbearing age in the United States. Given that adequate vitamin D concentrations in pregnant women are associated with healthy reproductive outcomes, periconceptional intervention programs may focus on raising vitamin D levels in these women and efforts to prevent vitamin D deficiency-linked health outcomes should begin during routine gynecologic care prior to conception and continue through the postpartum period. The multiple risk factors for vitamin D deficiency and inadequacy that we identified in this study should be useful in identifying at-risk groups of women who may be targeted by these programs. 

## Figures and Tables

**Table 1:** Unadjusted and adjusted prevalence ratios (with 95% confidence intervals)* for vitamin D deficiency and inadequacy among women aged 20–44 years, NHANES 2003–2006, *N* = 1,814.

Characteristic		Vitamin D deficiency		Vitamin D inadequacy	
	*N*	Unadjusted	Adjusted	Unadjusted	Adjusted
Demographic					
Age (yrs)					
20–27	651	1.00	1.00	1.00	1.00
28–34	540	0.98 (0.67–1.43)	1.02 (0.78–1.34)	0.94 (0.76–1.16)	0.92 (0.78–1.09)
35–44	623	1.17 (0.83–1.66)	1.19 (0.81–1.74)	1.09 (0.86–1.39)	1.10 (0.88–1.38)
Race/ethnicity					
Non-Hispanic white	856	1.00	1.00	1.00	1.00
Non-Hispanic black	370	12.82 (7.31–22.47)	8.27 (5.06–13.50)	3.21 (2.50–4.13)	2.78 (2.14–3.61)
Mexican American	417	5.47 (2.85–10.51)	4.14 (2.20–7.78)	2.48 (1.93–3.18)	1.78 (1.36–2.33)
Other	171	5.36 (2.72–10.56)	5.44 (3.03–9.78)	2.48 (1.89–3.24)	2.25 (1.74–2.92)
Education					
<High school diploma	393	1.00	1.00	1.00	1.00
High school graduate	383	0.69 (0.47–1.02)	1.06 (0.74–1.52)	0.78 (0.57–1.06)	1.03 (0.78–1.36)
>High school diploma	1,038	0.39 (0.29–0.55)	1.00 (0.75–1.34)	0.53 (0.42–0.66)	0.86 (0.67–1.10)

Pregnancy status					
Currently pregnant	435	0.73 (0.37–1.45)	0.72 (0.41–1.26)	0.66 (0.45–0.96)	0.70 (0.49–1.00)
With a history of pregnancy	1,034	1.38 (0.99–1.92)	0.82 (0.57–1.16)	1.21 (0.93–1.59)	0.89 (0.71–1.11)
Never pregnant	345	1.00	1.00	1.00	1.00

Diet and supplementation					
Milk consumption					
Never	222	1.00	1.00	1.00	1.00
>0 to <1 time/day	730	0.54 (0.39–0.76)	0.77 (0.57–1.06)	0.94 (0.67–1.32)	0.87 (0.64–1.18)
≥1 time/day	862	0.20 (0.13–0.34)	0.34 (0.23–0.51)	0.59 (0.42–0.83)	0.56 (0.40–0.77)
Fish consumption					
Never	573	1.00	1.00	1.00	1.00
>0 to <2 times/wk	1,134	0.79 (0.52–1.19)	0.75 (0.56–1.01)	1.00 (0.83–1.21)	1.01 (0.82–1.24)
≥2 times/wk	107	0.67 (0.33–1.34)	0.62 (0.31–1.23)	0.64 (0.40–1.03)	0.71 (0.48–1.05)
Dietary supplement use				
Yes	943	0.30 (0.22–0.40)	0.57 (0.42–0.78)	0.55 (0.46–0.66)	0.69 (0.58–0.81)
No	871	1.00	1.00	1.00	1.00

Examination period					
Nov. 1-Apr. 31	857	1.00	1.00	1.00	1.00
May 1-Oct. 31	957	0.37 (0.23–0.60)	0.51 (0.37–0.71)	0.52 (0.40–0.68)	0.58 (0.47–0.73)

Body mass index (kg/m2)**					
<25.0	785	1.00	1.00	1.00	1.00
25.0–29.9	455	1.32 (0.93–1.86)	1.06 (0.77–1.46)	1.48 (1.13–1.93)	1.25 (0.97–1.60)
≥30.0	574	3.37 (2.41–4.72)	1.74 (1.27–2.38)	2.11 (1.58–2.81)	1.94 (1.54–2.45)

Chronic condition					
Hypertension					
Yes	120	2.44 (1.72–3.46)	0.95 (0.67–1.34)	1.39 (0.99–1.96)	0.92 (0.64–1.31)
No	1,694	1.00	1.00	1.00	1.00
Diabetes					
Yes	44	2.06 (1.13–3.74)	1.52 (1.01–2.32)	1.59 (1.08–2.34)	1.02 (0.68–1.54)
No	1,770	1.00	1.00	1.00	1.00
Cardiovascular disease					
Yes	32	3.12 (1.90–5.14)	2.30 (1.31–4.04)	1.51 (0.97–2.33)	1.50 (0.96–2.34)
No	1,782	1.00	1.00	1.00	1.00
Asthma					
Yes	171	1.35 (0.94–1.92)	1.00 (0.73–1.37)	0.86 (0.59–1.26)	0.89 (0.67–1.19)
No	1,643	1.00	1.00	1.00	1.00

Lifestyle-related behavior					
Current Smoking					
Yes	385	1.25 (0.85–1.85)	1.40 (1.06–1.85)	1.06 (0.86–1.31)	1.08 (0.89–1.31)
No	1,429	1.00	1.00	1.00	1.00
Physical activity					
Yes	1,558	0.39 (0.28–0.54)	0.87 (0.61–1.24)	0.77 (0.60–0.99)	1.19 (0.92–1.54)
No	256	1.00	1.00	1.00	1.00
Any alcohol use					
Yes	1,203	0.52 (0.39–0.71)	0.82 (0.61–1.09)	0.69 (0.56–0.84)	0.90 (0.75–1.07)
No	611	1.00	1.00	1.00	1.00

Healthcare access					
Insurance coverage					
Yes	1,388	0.59 (0.41–0.84)	1.01 (0.70–1.46)	0.65 (0.52–0.80)	0.94 (0.77–1.15)
No	426	1.00	1.00	1.00	1.00
Healthcare visit					
Yes	1,609	0.61 (0.42–0.87)	0.87 (0.64–1.19)	0.70 (0.54–0.90)	0.84 (0.64–1.09)
No	205	1.00	1.00	1.00	1.00

*Prevalence ratios and 95% confidence intervals were estimated using log-linear regression models without and with adjustment for all other variables listed in the table; women with serum 25(OH)D ≥20 ng/mL was used as the referent.

**Self-reported prepregnant weight and height were used to calculate body mass index (BMI) for pregnant women, and measured weight and height were used to calculate BMI for nonpregnant women.
